# Does *TGFBR3* Polymorphism Increase the Risk of Silent Cerebral Infarction in Egyptian Children with Sickle Cell Disease?

**DOI:** 10.1007/s12098-022-04181-5

**Published:** 2022-07-04

**Authors:** Hoda Hassab, Marwa Hanafi, Ahmed Elbeheiry, Mona Hassan, Yasmine El Chazli

**Affiliations:** 1 Department of Pediatrics, Hematology, and Oncology Unit, Faculty of Medicine, Alexandria University, Alexandria, 21256 Egypt; 2 Department of Clinical and Chemical Pathology, Faculty of Medicine, Alexandria University, Alexandria, Egypt; 3 Department of Radiodiagnosis and Intervention, Faculty of Medicine, Alexandria University, Alexandria, Egypt; 4 Department of Pediatrics, Faculty of Medicine, Helwan University, Cairo, Egypt

**Keywords:** Sickle cell disease, Silent cerebral infarct, TGFBR3, MRI, Children

## Abstract

**Objectives:**

To evaluate the relationship between TGFBR3 rs284875 single nucleotide polymorphism (SNP) state and silent cerebral infarction (SCI) in asymptomatic patients with sickle cell disease (SCD).

**Methods:**

A cross-sectional study was conducted on 50 children with SCD above 2 y of age followed up at the hematology outpatient clinic of Alexandria University Children's Hospital in Egypt. Twenty-four healthy children were included as a control group. All patients included in the study were subjected to complete history and clinical examination. Real-time polymerase chain reaction was performed on patients and controls for identification of SNP rs284875 of the TGFBR3 gene. A magnetic resonance imaging (MRI) of the brain were performed only on patients for detection of SCI.

**Results:**

Fifty SCD patients were enrolled (26 males and 24 females), with a median age of 10.9 y (2.3–17.8 y), and 24 children as healthy control for the studied SNP. Thirty-five (70%) patients had homozygous SCD, while 30% had sickle β-thalassemia. The brain MRI was normal in all the patients except for 2 patients who had features of SCI. The TGFBR3 rs284875 SNP was detected in 15 (30%) patients in the homozygous state (GG) versus only 1 (4.2%) child from the control group (*p* = 0.003). The prevalence of SCI was low in the study population and there was no statistically significant relationship between the TGFBR3 rs284875 SNP status and the presence of SCI in the brain MRI (*p* = 0.621).

**Conclusions:**

This study confirmed a low prevalence of SCI in the SCD patient included in the study. The TGFBR3 rs284875 SNP did not significantly increase SCI among those patients.

## Introduction

Sickle cell disease (SCD) is the most common monogenic hemoglobin disorder. It is a multiorgan disorder associated with high morbidity, mortality, and poor quality of life. When deoxygenated, the abnormal hemoglobin “HbS” makes the erythrocytes sickle shaped, rigid, and prone to lysis, causing occlusion and vasculopathy [1]. About 25% of SCD patients may develop a neurological complication over their lifetime. Silent cerebral infarct (SCI) or “silent stroke”, is the most common form of neurologic injury among children with SCD [2, 3]. SCIs are more common in patients with hemoglobin SS (HbSS) but may also occur in individuals with heterozygous genotypes, including sickle β-thalassemia and HbSC disease [3]. Clinical risk factors for SCI include a history of seizures, low pain event rate, elevated white blood cell count, low baseline hemoglobin level, and intracranial stenosis [2, 4].

SCIs do not lead to apparent focal neurological symptoms or signs that might correlate with the lesion location absent on history and physical examination [5]. SCIs are identified incidentally or through screening of at-risk patients, using computed tomography scans, or ideally, magnetic resonance imaging (MRI) scans [6, 7]. SCIs in children with SCD are associated with an increased risk of future overt strokes and new or progressive SCIs [4]. Since primary prevention of vascular insults in SCD patients is of utmost importance, a number of studies have investigated possible genetic polymorphisms that may contribute to the vaso-occlusive process; only a few genetic factors are known to influence stroke risk. Five single nucleotide polymorphisms (SNPs) tested in children with SCD were significantly associated with stroke risk, namely ANXA2 (rs11853426), TEK (rs489347), and TGFBR3 (rs284875) variants, whereas ADCY9 (rs2238432) and ∝ -thalassemia were linked with decreased stroke risk [8].

The rs284875 SNP resides within the TGFBR3 gene, a transforming growth factor-β receptor (TGFBR) [9].The transforming growth factor‐β (TGF‐β) signaling pathway plays an important role in several cellular processes, regulating proliferation, migration, invasion, immune response, angiogenesis, vascular inflammation, hematopoiesis, and apoptosis. TGF‐β receptor III (TGFBR3) is a transmembrane proteoglycan that functions as a coreceptor of the TGF‐β superfamily [8, 10]. Stroke has been directly associated with variants in the TGFBR3 gene; further investigations of this gene may help define specific mutations that actually affect biological functions and confer stroke risk or protection in children with SCD [9, 10]. The aim of this work was to detect the frequency of the TGFBR3 rs284875 polymorphism and its relation to SCI assessed by brain MRI in asymptomatic patients with SCD.

## Material and Methods

This cross-sectional study was conducted from December 2018 to December 2019 and included 50 children with SCD (HbSS, HbSβ) above 2 y of age followed up at the hematology outpatient clinic of Alexandria University Children's Hospital in Egypt. This was a convenience sample as all patients SCD (and their parents) coming to the clinic for routine care were approached and were included if they agreed to participate. The study included most of the patients registered in the clinic, representing several Governorates of Northern Egypt. SCD was diagnosed by hemoglobin electrophoresis in all patients and confirmed by β-globin gene polymerase chain reaction (PCR) in 64% of patients (data retrieved from patients' files). Twenty-four healthy children were included as a reference group for the studied SNP, they were age and sex matched with the patients. After approval of the study by the "Alexandria University Ethical Committee” (IRB number 00012098), informed consent/assent was obtained from the patient's legal guardians/patients.

Patients were excluded from the study if they had abnormal neurologic symptoms or signs, a history of cerebral thrombotic events including overt stroke (ischemic or hemorrhagic), transient ischemic attacks, or seizures. All patients were subjected to a detailed history-taking with reviewing of medical records (for a period of 2 y), including:

-Frequency and severity of vaso-occlusive crises (VOC). VOC was defined as the persistence of pain in the extremities, head, chest, back, or abdomen for two or more hours that could not be explained except by the presence of SCD. The severity of VOC was considered mild if the patient had required or not pain medicines, but could do normal daily activity; as moderate when the patient requied pain medications and changes in daily activities, such as missing work or school. Severe VOC was used for pain episodes requiring a visit to the emergency department or physician's office or hospitalization [11].

-History of blood transfusion; chronic transfusion was defined by regular transfusions at four to 5-wk interval.

-Hydroxyurea (HU) intake, dose, duration, and compliance to treatment (by questioning parents).

A thorough clinical examination was done, including a complete neurological examination by a pediatric neurologist. Laboratory investigations included a complete blood count with a reticulocytes count, serum ferritin, hemoglobin electrophoresis. The identification of genetic variant (TGFBR3 rs284875 SNP), reported to be related with stroke in SCD [8, 10] was performed in patients and controls. Genomic deoxyribonucleic acid (DNA) was extracted from ethylenediaminetetraacetic acid—anticoagulated whole blood samples using QIAamp DNA Blood MiniKit (QIAGEN, Germany, Cat. no. 945105). TGFBR3 rs284875 SNPs genotyping were detected by 5' nuclease allele discrimination assay by real-time PCR using Stratagene real-time PCR system (QIAGEN, Germany).

Imaging studies included a brain MRI performed on a 1.5-T MRI scanner (Philips, Ingnia) using a multichannel head and neck coil. Criteria for acceptable images were checked by the study's single neuroradiologist. They included axial and sagittal spin-echo T1-weighted, coronal and axial turbo spin-echo T2-weighted, axial fluid-attenuated inversion-recovery images; DWI in the transverse plane, using a single-shot spin-echo echo-planar acquisition. Silent cerebral infarction has previously been defined as foci of abnormally elevated T2-weighted signal intensity on brain MRI that measure at least 3 mm in one or more axes and are seen in two imaging planes [3].

Nonparametric statistics were adopted, and analysis was performed using IBM SPSS package version 24. Qualitative data were expressed as number and percent. Quantitative data were described using the minimum and maximum, median, and the 25^th^ and 75^th^ percentiles expressed as the interquartile range (IQR). Hardy–Weinberg equilibrium (HWE) was assessed using the chi-square test (χ^2^ test). Univariate analysis, including chi-square and Monte Carlo tests, was used to test the significance of qualitative variables, while Kruskal–Wallis test was used for quantitative variables. The significance was judged at the 5% level.

## Results

The patient's demographic and clinical data are shown in Table 1. Thirty-five patients (70%) received HU drug therapy, with a median dose of 17 mg/kg (range 9–35 mg/kg); all children on hydroxyurea were compliant to the drug except 3 (8.6%) patients with a median duration of treatment of 5 y (range 1–13 y). Patients' investigations are shown in Table 2.

**Table 1:** Demographic and clinical characteristics of sickle cell disease patients

Patients’ characteristics	Sickle cell disease patients
	(*n* = 50) * N* (%)
Diagnosis	
Homozygous SCD	35 (70)
Sickle β-thalassemia	15 (30)
Sex	
Male	26 (52)
Female	24 (48)
Age at the time of the study (years)	
Min–Max	2.3–17.8
Median (IQR)	10.9 (7.5–14.1)
Age at the time of diagnosis (months)	
Min–Max	2.8–181.6
Median (IQR)	18.7 (10.9–44.7)
Transfusion regimen at the time of the study	
No transfusion	28 (56)
Regular transfusion regimen	8 (16)
Occasional transfusions	14 (28)
VOC frequency	
None	7 (14)
< 3 crises per year	5 (10)
≥ 3 crises per year	38 (76)
VOC severity	
None	7 (14)
Mild	10 (20)
Moderate	13 (26)
Severe	20 (40)

*IQR* Interquartile range, *Min–Max* Minimum–maximum, *n* Number of patients, *N* Number, *SCD* Sickle cell disease, *VOC* Vaso-occlusive crises

**Table 2:** Laboratory investigations for sickle cell disease patients (*n* = 50)

Laboratory investigations	Min–Max	Median (IQR)
*Complete blood picture parameters*		
Hemoglobin (g/L)	6.6**–**10.7	8.5 (7.8**–**9.1)
Total leukocyte count (× 10^9^/L)	3.5**–**22.7	10.2 (7.5**–**14.6)
Platelet count (× 10^9^/L)	125**–**1150	391.5 (257.8**–**541.3)
Mean corpuscular volume (fL)	53.5**–**109.6	86.6 (73.8**–**93.7)
Reticulocytes (%)	0.6**–**33.1	5.8 (2.8**–**10.3)
*Serum ferritin* (ng/dL)	38.2–6,500	622.4 (202.3–1,540.8)
Hemoglobin electrophoresis^*^		
Hemoglobin F (%)	0.0**–**35.7	8.7 (3.3**–**17.9)
Hemoglobin S (%)	26.2**–**94.4	69.6 (55.7**–**83.3)

^*^at time of the study

*Hemoglobin F* Fetal hemoglobin, *Hemoglobin S* Sickle hemoglobin, *IQR* Interquartile range, *Min–Max* Minimum*–*maximum

The polymorphism was not found in HWE among patients but was found in HWE among the reference group, as shown Table 3. The TGFBR3 rs284875 SNP analysis and comparison with different clinical and laboratory parameters are shown in Tables 4 and 5.

**Table 3:** Hardy–Weinberg equilibrium for TGFBR3 polymorphism

Genotype frequencies	Hardy–Weinberg equilibrium		Chi square test
	Observed no.	Expected no.	(*p* value)
Patients			
Homozygote reference (AA)	4	7.61	
Heterozygote (AG)	31	23.79	*X*^*2*^= 4.592
Homozygote variant (GG)	15	18.61	*p* = 0.1006
Variant allele frequency	0.61	50	
Reference group			
Homozygote reference (AA)	8	10.01	
Heterozygote (AG)	15	10.98	*X*^*2*^ = 2.115
Homozygote variant (GG)	1	3.01	*p* = 0.347
Variant allele frequency	0.35	24	

A *p* value ≥ 0.05 is consistent with Hardy–Weinberg equilibrium, *X*^*2*^ for chi-square test

*A* Major allele; *G* Mutant allele

**Table 4:** Results of rs284875 TGFBR3 analysis in patients and reference group

		Patients	Reference group	Test of significance	Adjusted odds ratio (CI)^**^
		*n* = 50	*n* = 24	*p* value	
		*N* (%)	*N* **(%)**		
TGFBR3	AA	4 (8)	8 (33.3)	*X*^*2*^= 11.424	Reference value
	AG	31 (62)	15 (62.5)	*p* = 0.003*	4.133 (1.07–15.93)
	GG	15 (30)	1 (4.2)		
Allele frequency	G	61 (61)	17 (35.4)	*X*^*2*^ = *8.515*	30.0 (2.85–315.61)
	A	39 (39)	31 (64.6)	*p* = 0.003*	

^*^Statistically significant at *p* < 0.05, ^**^Multinomial logistic regression model, *X*^*2*^ for chi-square test

*A* Major allele, *G* Mutant allele; *CI* Confidence interval, *n* Number of patients, *N (%)* Number (percent)

**Table 5:** Relation between TGFBR3 rs284875 SNP and some clinical parameters

	TGFBR3 rs284875 analysis			*p* value
	AG	AA	GG	
	>(*n* = 31)	(*n* = 4)	(*n* = 15)	
	*N* (%)	*N* (%)	*N* (%)	
SCI on MRI				
Yes	2 (100)	0 (0)	0 (0)	*p* = 0.621
No	29 (60.4)	4 (8.3)	15 (31.4)	
Diagnosis				
Homozygous SCD	24 (77.4)	1 (25)	10 (66.7)	*p* = 0.090
Sickle-thalassemia	7 (22.6)	3 (75)	5 (33.3)	
VOC frequency				
None	6 (24)	0 (0)	1 (6.7)	*p*^*MC*^ = 0.504
< 3 crises per year	6 (24)	0 (0)	1 (6.7)	
≥ 3 crises per year	21 (52)	4 (100)	13 (86.6)	
VOC severity (*n* = 40)				
Mild	6 (24)	0 (0)	4 (28.6)	*p*^*MC*^ = 0.343
Moderate	6 (24)	3 (75)	4 (28.6)	
Severe	13 (52)	1 (25)	6 (42.8)	
Hemoglobin (g/L)				
Median (IQR)	8.5 (7.8–9.5)	9.8 (8.2–10.7)	8.3 (7.8–8.8)	*p = *0.001^*^
Min–Max	6.6–10.5	8.0–10.7	6.7–10.7	
Reticulocytes (%)				
Median (IQR)	3.3 (2.2–7.9)	3.6 (3.5–4.9)	10.0 (7.3–19.0)	*p* = 0.001^*^
Min–Max	0.6–23.3	3.5–5.3	4.4–33.1	
Hb F at time of study				
Median (IQR)	7.3 (0.4–14.9)	15.5 (4.7–32.7)	9.8 (4.7–17.5)	*p* = 0.485
Min–Max	0.0–35.7	3.9–35.7	0.0–31.0	

^*^Statistically significant at *p* < 0.05

Kruskal–Wallis test to compare medians, *MC* Monte Carlo test, χ^2^ test for qualitative data

*A* Major allele, *G* Mutant allele, *IQR* Interquartile range, *Max* Maximum, *Min* Minimum, *MRI* Magnetic resonance imaging, *N (%)* Number (percent), *SCD* Sickle cell disease, *SCI* Silent cerebral infarction, *VOC* Vaso-occlusive crises

It was found that only 2 (4%) had features of SCI. Infarcts occurred in the deep white matter between the anterior cerebral artery and middle cerebral artery territories. Some incidental findings were noted and judged to be unrelated to the SCD. One patient had a small subcortical developmental venous anomaly (DVA) in the right occipital region. A partially empty sella turcica (ESS) was found in 2 patients. An unusual finding was detected, as hyperintense foci were noted on both corona radiata and centrum semiovale, notably on the left side likely related to Virchow–Robin space (VRS) in 1 patient. A bifid pituitary stalk was observed in 1 patient and pansinusitis in 2 patients. Agenesis of the corpus callosum (CC) was noted in 1 patient.

## Discussion

Increasingly, SCIs are recognized as a prevalent and progressive cerebrovascular complication in children with SCD and cognitive impairment, but they may be challenging to detect on routine screening by MRI [3]. Moreover, before 7 y of age, this usually requires sedation or general anesthetic, which carries significant risks in children with SCD and is unsuitable for screening. MRI is also not widely available in many African countries, where SCD is most prevalent [12]. Hence, assessing the prevalence of SCI in the present patient population to determine its value as a routine screening tool for SCD children was important.

Several candidate genetic polymorphisms have been proposed to affect stroke risk, but few have been validated. However, all of these validated SNPs are intronic, and it is not known if they directly affect stroke risk or if they may be linked to other nearby genetic polymorphisms that actually modify the stroke risk. Further investigations at these genetic regions may help define specific mutations that affect biological functions and confer stroke risk or protection in children with SCD [10]. Among SNPs linked to stroke risk in SCD patients, the rs284875 SNP residing within the TGFBR3 gene has been linked with cerebrovascular disease [8, 10]. Therefore, 50 children with SCD were tested to evaluate the TGFBR3 rs284875 SNP's effect in the present population.

In the current study, the prevalence of SCI was 4%, which represents a low prevalence; the 2 affected patients were HbSS. There are several reports for the incidence of SCI in SCD, as Lotfy et al. [13] found that the incidence was 3 (15%) patients among 20 Egyptian patients with SCA with a mean age of 9.5 ± 3.1 y. Mourad et al. [14] found that the incidence was 2 (20%) patients among 20 Egyptian patients with SCA. Tantawy et al. [15] showed that 5 (16.7) patients among 30 Egyptian patients with SCD aged less than 18 y. Similarly, in a French cohort of 173 children (5–15 y) with SCD, the silent stroke rate was 15% (15.2% of HbSS patients) [16]. Strumph et al. [17] reported that 7 (17%) of SCD patients had SCIs, 42 children with hemoglobin SS, hemoglobin SC, or hemoglobin Sβ^0^, between the ages of 5 and 21 y, were included in the study. Another study conducted on 224 Tanzanian children aged five to 19 y with SCA showed that prevalence of SCI was 29% [18, 19]. Pegelow et al. [19] found that up to 37% of their patients with SCD (HbSS or sickle-Sβ^0^), aged 2 and 17 y, had silent infarction. Similar findings were reported by Tewari et al. [12] as 19 (37%) patients with SCA (HbSS), aged 8–18 y, had SCIs; 7 patients with SCI were on HU therapy.

The variation in the incidence of SCI among different studies may be due to different and usually small sample sizes, and different treatment regimens, as it has been documented that chronic blood transfusion reduces the risk of SCI and acute stroke. The STOP study compared stroke rates in 63 children who received periodic transfusion with 67 children who were getting standard supportive care; after 1 y, 10 children in the standard care group had a stroke, while only 1 child in the transfusion group suffered a cerebral infarction [20]. Pegelow et al. [19] also found that among their patients with SCD, those receiving standard care were more likely to develop new silent lesions or stroke than those who received transfusions.

Some unusual MRI findings were detected in the present study; 1 patient showed dilated Virchow–Robin spaces (dVRS) which are defined as cerebrospinal fluid-like signal lesions (hypointense on T1 and hyperintense on T2) of round, ovoid, or linear shape with a maximum diameter of 3 mm, having smooth delineated contours, and located in areas supplied by perforating arteries. For lesions fulfilling the same criteria, except for having a diameter of more than 3 mm, further efforts are needed to differentiate them from infarcts [21]. Zhu et al. [22] concluded that dVRS should be considered another MRI marker of cerebral small vessel disease and stroke risk.

In another patient, there was agenesis of the CC. The reduction in the size of the CC with cerebral infarction would be expected given the known effects of cerebral tissue injury on interhemispheric axons [23]. Schatz et al. [24] reported a decreased CC size among children with SCD, even in the absence of visible infarcts in this region. Balci et al. [25] reported that there was noticeable diffuse atrophy in the CC paralleling the brain atrophy seen in almost all SCD patients than the healthy control. ESS is the condition when the pituitary gland shrinks or becomes flattened, filling the sella turcica with cerebrospinal fluid instead of the normal pituitary. In the present study, two patients showed partially ESS. Soliman et al. [26] reported that SCD is an important cause of ESS, especially in children with retarded growth. This could be due to ischemia/atrophy of the pituitary gland following one or more sickling attacks. Other accidentally discovered congenital anomalies have no clinical significance.

In the present study, most patients (62%) were heterozygotes (AG) for TGFBR3 rs284875 SNP, while 30% were homozygote for the minor allele (GG), which was more prevalent than in the reference group (*p* = 0.003). Santiago et al. [27] reported that 17.1% of cases (30/175) were heterozygotes (AG), while 80.6% (141/175) were homozygote for the minor allele (GG). In the current study, TGFBR3 rs284875 SNP, not associated with SCI risk, may be because only 2 children had SCI. In contrast, Belisário et al. [28] showed that the TGFBR3 rs284875 SNP was associated with high-risk TCD but not with acute cerebral ischemia risk. Several other studies also showed that the TGFBR3 rs284875 SNP had been linked with increased stroke risk [10, 29, 30].

The current study showed a significantly lower hemoglobin level in patients with the mutant allele G of the TGFBR3 rs284875 SNP, while having a significantly higher reticulocyte count. However, there was no statistically significant relationship between the mutant allele G of the TGFBR3 rs284875 SNP and the hemoglobin F percent or the VOC frequency and severity. Similarly, Santiago et al. [27] showed no significant association between TGFBR3 rs284875 SNP and clinical manifestations in SCD as VOC or stroke.

This study had several limitations, first was the small number of patients found to have SCI by MRI, which made the identification of risk factors unlikely. Second, only one SNP related could be tested to increased stroke risk due to financial constraints. Finally, the study was cross-sectional, and the absence of SCI at the time of the study does not mean that some of the patients will not develop some later, hence, the importance of a longitudinal routine follow-up of these patients. Nevertheless, this study has some strengths; first, it represents the starting point of assessment of neurological sequelae in a vulnerable population and raises awareness for the importance of instituting regular follow-up program for these patients. Second, it opens the door to a multidisciplinary teamwork and should prompt further prospective and multicenter/national studies.

## Conclusion

This study confirmed a low prevalence of SCI in the present SCD patient population. The study had some limitations and confounding factors, as transfusions and hydroxyurea therapy. Nevertheless, the TGFBR3 rs284875 SNP was more common among SCD patients than in normal controls, and it did not significantly increase SCI among children with SCD.

## Data Availability

On reasonable request.
